# Treatment and prognosis analysis of patients with moderate-volume hypertensive basal ganglia haemorrhage using DTI-guided stereotactic puncture-based surgery

**DOI:** 10.3389/fneur.2025.1619514

**Published:** 2025-07-31

**Authors:** Jinhong Li, Hu Zhou, Jie Li, Junyi Shu, Shiqiang Yang, Anqiang Yang

**Affiliations:** ^1^Department of Neurosurgery, The First People’s Hospital of Yibin, Yibin, Sichuan, China; ^2^College of Medicine and Life Sciences, Chengdu University of Traditional Chinese Medicine, Chengdu, Sichuan, China; ^3^College of Clinical Medicine, Chengdu Medical College, Chengdu, Sichuan, China

**Keywords:** hypertensive basal ganglia haemorrhage, stereotactic puncture drainage, moderate-volume, diffusion tensor imaging, assessment of daily living

## Abstract

**Background:**

Hypertensive basal ganglia haemorrhage (HBGH) is a prevalent critical condition in neurosurgery characterised. Severe neurologic dysfunctional outcome despite systemic treatment. The objective of this study is to examine the impact of stereotactic minimally invasive puncture and drainage utilising DTI on the efficacy and quality of life of patients diagnosed with moderate-volume HBGH.

**Method:**

Statistical analysis was performed on variables related to each group and clinical prognosis. The primary outcomes of the study were the Glasgow Outcome Scale (GOS) and Activities of Daily Living (ADL) scores 6 months after treatment commenced. Linear regression analysis was used to evaluate the risk factors influencing these outcomes. Multivariate regression modelling was then used to compare the impact of the different treatment modalities on the primary outcome in the three patient groups. Finally, sensitivity and subgroup analyses were performed to verify the stability of the study findings.

**Results:**

This retrospective study enrolled 65 patients with moderate-volume basal ganglia haemorrhage following a rigorous screening process. The group was divided into a conservative group, a conventional stereotactic group and a DTI-guided stereotactic group according to the main treatment modality. At 12 h, 48 h, 7 days, and 2 weeks after treatment, the amount of residual hematoma was significantly lower in both surgical groups than in the conservative group (*p* < 0.001). Both surgical groups had significantly higher ADLs than the conservative group after 6 months of treatment (All *p* < 0.05). In linear multifactorial regression analysis, the conventional stereotactic group (*β* = 17.82, *p* = 0.003) and the DTI-guided stereotactic group (β = 35.33, *p* < 0.001) had higher ADL scores with statistically significant differences compared with the conservative treatment group.

**Conclusion:**

In patients with moderate-volume hypertensive basal ganglia cerebral haemorrhage, those treated with DTI-assisted stereotactic surgery may exhibit superior long-term neurological recovery compared to those managed with medical conservative treatment or conventional stereotactic surgery.

## Introduction

Hypertensive intracerebral haemorrhage (HICH) is a serious, life-threatening condition associated with hypertension. The aetiology and causative factors of HICH are generally considered to be poorly controlled blood pressure, large fluctuations in blood pressure, persistent hypertension, atherosclerosis, amyloid angiopathy, fibrous glass-like lesions of cerebral vascular walls, emotional stress, and genetic factors (1). Dietary changes, hyperlipidemia, diabetes mellitus, and obesity have also been identified as significant risk factors for cerebrovascular disease caused by HICH (2). In recent years, the incidence of HICH has been increasing on an annual basis, accounting for approximately 70 to 80% of spontaneous cerebral haemorrhages (3). The mortality rate within 30 days is as high as 35 to 52%, and most of these cases are accompanied by serious disability (4), which places a significant economic and mental burden on patients and their families (5). The basal ganglia region is a frequent haemorrhage site in HICH, accounting for 50 to 70% of all spontaneous intracranial haemorrhage (6). The region’s small volume and proximity to significant functional centres, including limb sensory-motor nerves and the brainstem, are of particular concern. Which has a high lethality and disability rate, and survivors often face a range of physical disabilities and impaired higher neurological functions, such as speech and cognitive abilities (7). In recent years, as the population in China has aged, the number of patients suffering from hypertensive cerebral haemorrhage has increased (8). Although the incidence is highest in the middle and older age groups, there has been a gradual rejuvenation in recent years, which has had a serious impact on patients’ daily lives and ability to work (9, 10). There is no clinical consensus regarding the optimal treatment approach for patients with HICH in the basal ganglia region, presenting with a haemorrhage volume of 20–40 mL and no evidence of cerebral herniation.

The basal ganglia region is the most common site for HICH. This is due to the depth of the lesion and the large number of peripheral nerve nuclei and bundles in the region. Hematomas at this site often result in impaired consciousness and significant neurological damage. Conventional surgery may cause further damage and permanent neurological deficits. Therefore, conservative treatment is often the preferred option for physicians, conscious patients, and patients being treated in a clinical setting. Consequently, even patients with moderate bleeding and stable vital signs may experience severe neurological deficits following systemic treatment. Although conservative drug therapy helps to resorb the haematoma, prolonged compression and haematotoxicity may exacerbate cerebral oedema. Therefore, it is crucial to remove the haematoma as quickly as possible when treating this group of patients.

With the iterative updating of neuroimaging technology, the development of microsurgical instruments, and the application of precise surgical strategies, there is an increasing number of systematic studies of HICH that provide a more adequate evidence-based basis for clinical diagnosis and treatment decisions. Diffusion tensor imaging (DTI) is currently recognised as the most attractive non-invasive test. The use of DTI in patients with cerebral haemorrhage can identify the location of hematomas in relation to the corticospinal tract (CST) (11). The spatial relationship of the hematoma to the corticospinal tract can be accurately visualised in three dimensions, avoiding potential damage to the CST during surgery (12). In recent years, the development of stereotactic minimally invasive surgery has opened a new way for patients with HICH (13). In this study, we mainly compared the clinical efficacy and its prognosis of DTI-assisted stereotactic puncture and drainage surgery, conventional stereotactic puncture and drainage surgery, and internal conservative treatment in patients with moderate-volume cerebral haemorrhage, in order to provide important treatments, surgical modalities, and technological ways for clinical decision-making, and to search for a safer and more effective therapeutic option that will further improve patient efficacy and prognosis.

## Methods

### Study design and population

**Figure 1:**
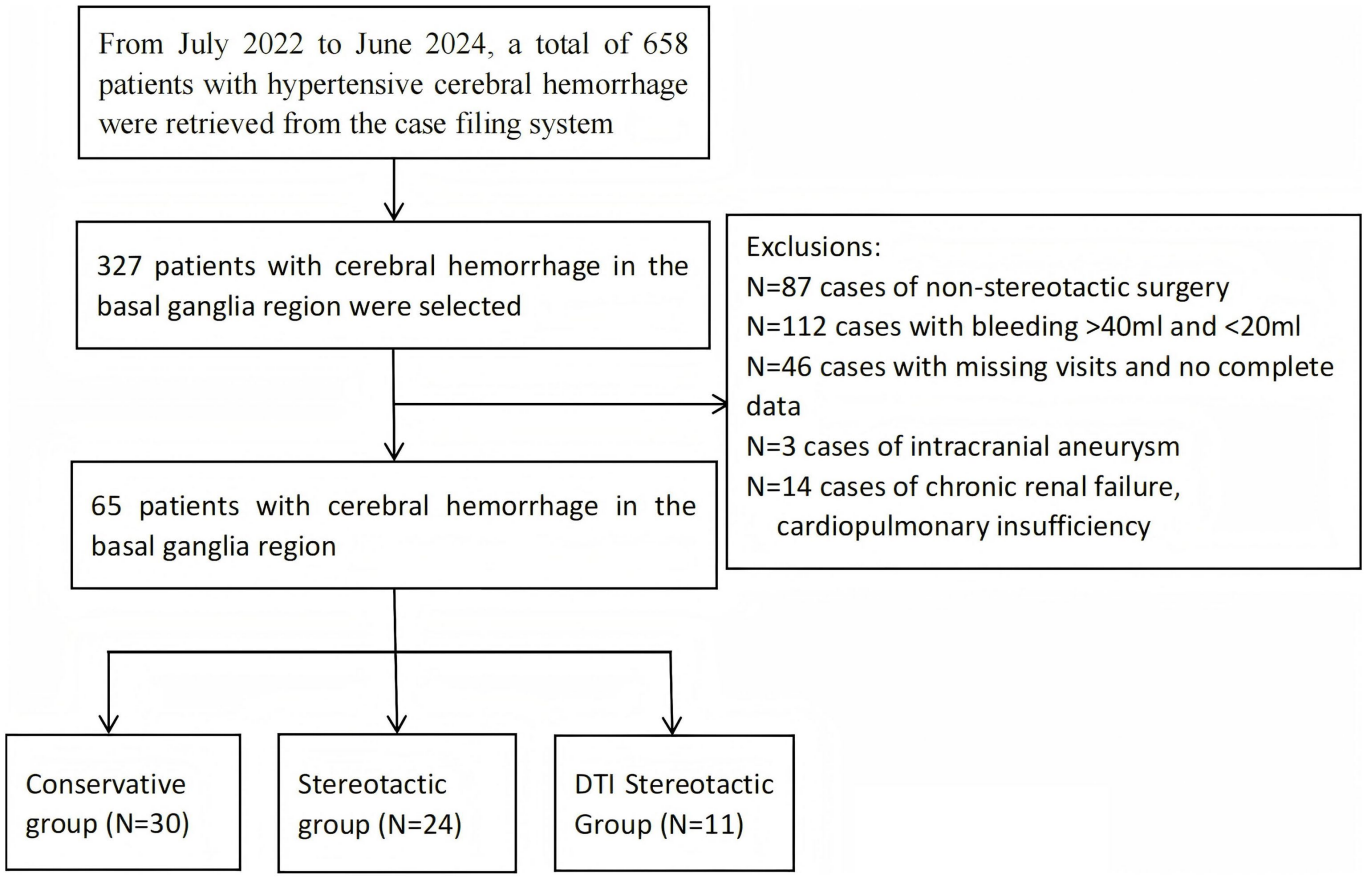
Flow chart. This flowchart illustrates the screening and grouping process for patients with hypertensive cerebral haemorrhage who participated in the study.

This study retrospectively selected 65 patients with HBGH at the Department of Neurosurgery, First People’s Hospital, Yibin City, Sichuan Province, China, from October 2022 to June 2024. As this was a retrospective study, informed consent was not required. These patients were selected according to the following selection criteria. Inclusion criteria included the following:(1) meeting the diagnostic criteria for hypertensive cerebral haemorrhage (accurate history of hypertension, with hypertension defined as systolic blood pressure ≥140 mmHg and/or diastolic blood pressure ≥90 mmHg); (2) the patient was admitted to hospital and assessed by cranial CT scan, the site of the hematoma was located in the basal ganglia region and the volume of the hematoma ranged from 20 to 40 mL; (3) onset of illness ≤ 24 h prior to admission and no previous history of cerebral haemorrhage; (4) normal coagulation function. Exclusion criteria included: (1) patients with haemorrhage volume greater than 40 mL, cerebral herniation and bilateral dilated pupils on admission; (2) patients with secondary causes of cerebral haemorrhage (e.g., craniocerebral trauma, cerebral vascular structural abnormality, aneurysm rupture, stroke from intracranial tumour or hemorrhagic cerebral infarction, etc.); (3) associated with serious underlying diseases such as major organ (heart, liver, lung and kidney) or blood system failure; (4) with a history of previous brain surgery or severe traumatic brain injury; (5) meeting the clinical diagnostic criteria for brain death at the time of admission (Figure 1).

All subjects were identified through the hospital case management system. Subjects were then divided into three treatment groups based on the primary method used during the period. These were the conservative treatment group (30 cases), group A (Conventional stereotactic,24 cases) and group B (DTI-guided stereotactic,11cases). Patient sex, age, smoking and alcohol history, volume and location of haemorrhage, admission GCS score, NIHSS score, admission blood pressure, history of hypertension, history of diabetes mellitus, history of coronary artery disease, in-hospital complications, length of hospital stay, and laboratory indices were collected; as well as post-treatment information such as 2-week GCS scores and amount of residual hematoma at 12 h, 48 h, 7 days, and 2 weeks after treatment. Finally, GOS and ADL scores at 6 months post-discharge were obtained from all patients through on-site and telephone follow-up and recorded in the database as primary outcomes.

The following factors were considered when selecting the 11 patients for DTI-guided technology: (1) the location of the haematoma in relation to important functional areas, such as the corticospinal tract (CST). DTI-guided technology can more accurately avoid these areas, thereby reducing the potential damage to neurological function caused by surgery.(2) The patients were in good general health, enabling them to tolerate more complex preoperative preparations and surgical procedures.(3) Patients or their families were highly accepting of the new technology. These factors collectively led us to choose DTI-guided technology for surgery.(4)Since our research group received project funding in 2023 and gradually began this research project, the number of cases in this group of surgeries has been relatively small.

### Treatment process

In the preoperative imaging evaluation phase of DTI stereotactic puncture, the patient first received a DTI scan, and then the frame locator of the brain stereotactic device was fixed to the skull via the scalp under local anaesthesia, and a CT scan was performed, and the raw data of DTI and CT were imported into the stereotactic visualisation and localisation software, respectively, and the three-dimensional stereoscopic model of the cranial bone structure, the area of the intracranial hematoma and the CST were reconstructed by the software to understand their spatial relationship. Then, the target for puncture was set at the centre of the largest plane of the hematoma under visualisation conditions, avoiding the CST and major blood vessels, and the puncture path, angle and depth were simulated, while the distance between the scalp and the target was measured. The sample case of ICH, shown in Supplementary Figure 1 and incorporating DTI, examined the relationship between the haemorrhage and the corticospinal tract after visualisation. The design of the preoperative puncture access and the postoperative review were also examined. Extraction was generally completed within 7 days to minimise the risk of intracranial infection.

In the conventional stereotactic group, patients were admitted to hospital for preoperative preparation. Stereotactic frame fixation was performed under local anaesthesia, followed by CT scanning. The CT thin-slice scan data were then imported into stereotactic visualisation and localisation software for image alignment and spatial correction in order to reconstruct a three-dimensional model of the skull and intracranial haematoma. ‘The puncture target was set at the centre of the largest plane of the haematoma. Ventricular structures and the distribution of functional areas were considered to avoid vital areas. Finally, the optimal puncture trajectory along the long axis of the haematoma was selected to simulate the surgical target and puncture path’. Although urokinase has been reported to have neuroprotective effects in cases of intracerebral haemorrhage, it may also increase the risk of rebleeding and postoperative infection. Therefore, we did not administer urokinase to the surgical group once most of the haematoma had been removed. Both surgical groups achieved good results in terms of haematoma removal without the use of urokinase.

Patients in the conservative treatment group were admitted to hospital for continuous cardiac monitoring and were routinely treated for hypotension, dehydration, haemostasis, nutritional support, maintenance of internal environment and prevention of complications, with attention to level of consciousness, pupillary reflex to light and limb mobility, with vigilance for progressive neurological deterioration suggesting the possibility of cerebral herniation. In general, the first cranial CT scan is routinely performed within 24 h of admission and serves as a baseline for comparison of subsequent treatments. According to clinical progress, the antihypertensive and dehydration treatment plan should be adjusted, and then the CT scan should be reviewed according to the needs of the condition, which may be extended to weekly joint cephalothoracic CT evaluation during the stabilisation period.

Establish a procedure for quantitative analysis of the CT image to calculate the volume of the intracranial hematoma by reviewing the cranial CT at 12 h, 48 h, 7 days and 2 weeks after the onset of the illness.

### Outcome evaluation

The primary outcomes of this study were the GOS and ADL scores, which were used to assess patients’ ability to perform activities of daily living and neurological recovery after 6 months of treatment. Based on previous academic work, patients were considered to have severe neurological dysfunction if they scored less than 40, whereas patients with scores equal to or greater than 40 were considered to have significant treatment benefit (14). Secondary outcomes included assessment of hematoma resolution at 12 h, 48 h, 7 days and 2 weeks post-treatment, as well as GCS scores at 2 weeks post-treatment and complications during hospitalisation in both groups.

### Statistical analysis

The study was analysed retrospectively, with all primary data collected via medical record report forms. An independent statistician reviewed and optimised the statistical analysis for this study. Continuous variables were expressed as the mean ± standard deviation (SD) or the median and interquartile range (IQR), while categorical variables were expressed as percentages. Categorical variables were analysed using the chi-squared test or Fisher’s exact test to determine whether there were any differences between the three groups, while continuous variables were analysed using a t-test. Additionally, one-way linear multifactorial regression analyses were performed to assess differences in the main observations between the three groups. Finally, sensitivity analyses, such as subgroup analyses, were performed as necessary to verify the stability of the results. All statistical analyses were performed using the R 4.3.2 statistical package (http://www.R-project.org, The R Foundation, Shanghai, China) and Free Statistics V1.9 (Wind Rush Statistics) software. Study correlations were assessed using regression coefficients (*β*) and 95% confidence intervals (95% CI), with *p* < 0.05 being considered statistically significant.

## Results

### Basic characteristics

According to the strict inclusion and exclusion criteria in the flow chart, a total of 65 patients were enrolled in this study, including 51 males and 14 females with a mean age of 59.6 ± 10.8 years. Thirty (46.2%) of the patients had a history of smoking and 25 (38.5%) had a history of alcohol consumption. The mean time from onset to admission was 6.1 ± 2.1 h for all patients. The mean volume of haemorrhage in all patients on admission was 27.0 ± 5.3 mL, and the site of haemorrhage was the left basal ganglia region in 33 (50.8%) of the patients, and 11 (16.9%) had a combined hematoma infiltrating the ventricle. The mean admission NIHSS score for all patients was 14.1 ± 5.0 and the mean admission GCS score was 10.2 ± 1.7. There was no significant difference in the baseline characteristics of the patients in each group, *p* > 0.05, and they were comparable. Detailed information on the baseline characteristics of the patients is given in Table 1.

**Table 1:** Baseline characteristics of the study participant.

Variables	Total	Conservative treatment	Conventional stereotactic	DTI-guided stereotactic	*p*
	(*n* = 65)	(*n* = 30)	(*n* = 24)	(*n* = 11)	
Baseline characteristics					
Age (years)	59.6 ± 10.8	60.0 ± 9.8	59.5 ± 11.4	58.5 ± 13.1	0.923
Gender, Male (%)	51 (78.5)	23 (76.7)	20 (83.3)	8 (72.7)	0.798
ODTT (h)	6.1 (1.2,9.5)	5.5 (1.1,9.4)	5.6 (1.3,9.6)	5.9 (1.6,9.7)	0.221
BVA (mL)	27.0 ± 5.3	25.6 ± 4.3	27.1 ± 5.3	30.5 ± 6.8	0.029
Hematoma site, LBG (%)	33 (50.8)	13 (43.3)	14 (58.3)	6 (54.5)	0.528
HRIV, Yes (%)	11 (16.9)	5 (16.7)	4 (16.7)	2 (18.2)	1
Score system, points					
NIHSS (on admission)	14.1 ± 5.0	12.8 ± 4.4	15.1 ± 5.7	15.5 ± 4.4	0.161
GCS (on admission)	10.2 ± 1.7	10.5 ± 1.7	10.0 ± 1.7	9.8 ± 1.9	0.427
Risk factors					
Smoking, Yes (%)	30 (46.2)	10 (33.3)	13 (54.2)	7 (63.6)	0.138
Drinking, Yes (%)	25 (38.5)	10 (33.3)	10 (41.7)	5 (45.5)	0.705
Laboratory tests					
WBC (×10^9^/L)	9.8 ± 3.0	9.2 ± 3.1	9.8 ± 3.0	11.3 ± 2.4	0.124
Neutrophil (×10^9^/L)	8.3 ± 3.0	7.8 ± 3.2	8.3 ± 2.9	9.7 ± 2.2	0.173
PT (s)	13.7 ± 0.9	14.0 ± 0.6	13.4 ± 1.0	13.4 ± 1.0	0.029
APTT (s)	29.4 ± 4.1	29.0 ± 2.4	29.5 ± 5.2	30.4 ± 5.1	0.631
Serum albumin (g/L)	40.0 ± 4.1	40.5 ± 3.2	40.2 ± 3.8	38.4 ± 6.2	0.346
Urea nitrogen (mmol/L)	5.2 ± 2.5	5.0 ± 1.8	5.4 ± 3.3	5.5 ± 2.3	0.83
Potassium (mmol/L)	3.7 ± 0.5	3.6 ± 0.5	3.7 ± 0.5	3.8 ± 0.4	0.366
Sodium (mmol/L)	138.7 ± 3.6	138.5 ± 3.0	138.5 ± 4.1	139.7 ± 4.0	0.592
Calcium (mmol/L)	2.2 ± 0.3	2.3 ± 0.4	2.1 ± 0.1	2.2 ± 0.2	0.226

ODTT, Onset of disease-treatment time; GCS, Glasgow Coma Score; NIHSS, National Institutes of Health Stroke Scale; BVA, Bleeding volume on admission; LBG, Left basal ganglia; HRIV, Hematoma ruptured into ventricle; WBC, White blood cell; PT, Prothrombin time; APTT, Activated partial thromboplastin time.

### Assessment of prime results

**Figure 2:**
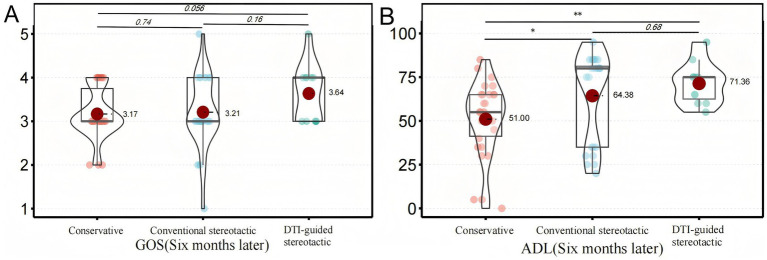
Analysis of prime results. This shows how the primary outcomes of the different treatment groups compare after six months. Panel **(A)** illustrates the distribution of GOS scores among patients in each group. There was no statistically significant difference between the groups. Panel **(B)** shows the distribution of ADL scores for each group of patients. A significant difference was observed between the conservative treatment group and the conventional stereotactic group (^*^p < 0.05). A significant difference was also observed between the conservative treatment group and the DTI-guided stereotactic group (^**^p < 0.01).

The primary outcome of this study was the GOS and ADL scores of the three groups of patients at follow-up after 6 months of treatment (10). The 6-month ADL scores of the patients in the DTI-guided stereotactic (group B) were (71.4 ± 11.9), in the conventional stereotactic group (group A) they were (64.4 ± 25.8) and in the conservative group they were (51.0 ± 21.1), which is a statistically significant difference (*p* < 0.001) (Figure 2). Based on the definitions in previous literature, in this study we categorised patients with ADL scores below 40 as having severe neurological deficits. 16 out of 65 patients had severe neurological deficits, representing 24.6% of all patients. DTI-guided stereotactic group had the lowest incidence of severe neurological deficits, 9.1%, with 1 case out of 11 patients. In contrast, conventional stereotactic group had a severe neurological deficit rate of 29.2%, with 7 out of 24 patients. The rate of severe neurological deficits in the conservative treatment group was 26.7%, with 8 out of 30 patients. In the GOS score of this study, there was 1 death, 5 cases of vegetative survival status, 37 cases of severe disability, 20 cases of mild disability and 2 cases of good recovery in 65 patients. The prognostic distribution was dominated by grade 3 (moderate disability) and grade 4 (mild disability), with no significant difference between groups (*p* = 0.576) (Table 2).

**Table 2:** Comparison of outcomes during hospitalisation in each group.

Variables	Total	Conservative treatment	Conventional stereotactic	DTI-guided stereotactic	*p*
	(*n* = 65)	(*n* = 30)	(*n* = 24)	(*n* = 11)	
HRV,12 h, Median (IQR)	20.1 (9.3, 23.6)	24.1 (22.5, 28.2)	9.7 (6.2, 15.4)	9.4 (6.2, 11.1)	**<0.001**
HRV,48 h, Median (IQR)	17.8 (6.2, 22.9)	24.1 (21.8, 27.9)	6.4 (3.6, 11.7)	7.2 (3.7, 9.9)	**<0.001**
HRV,7d, Median (IQR)	12.1 (2.4, 18.0)	18.1 (16.8, 22.3)	2.3 (1.3, 10.1)	2.8 (2.2, 4.8)	**<0.001**
HRV,2 W, Median (IQR)	4.8 (0.0, 12.0)	12.3 (9.1, 14.7)	0.3 (0.0, 3.7)	0.7 (0.0, 1.2)	**<0.001**
GCS,2 W, SD	11.3 ± 2.0	11.7 ± 1.8	10.8 ± 2.1	11.3 ± 2.3	0.216
DOH, days	27.5 ± 10.0	28.4 ± 7.9	25.4 ± 11.6	24.5 ± 11.5	0.425
GOS (6 months later)	3.3 ± 0.7	3.2 ± 0.6	3.4 ± 0.8	3.6 ± 0.7	0.125
ADL (6 months later)	59.4 ± 23.0	51.0 ± 21.1	64.4 ± 25.8	71.4 ± 11.9	**0.015**
Lung infections, *n* (%),yes	51 (78.5)	21 (70)	20 (83.3)	10 (90.9)	0.323
Gastrointestinal bleeding, *n* (%),yes	21 (32.3)	6 (20)	8 (33.3)	7 (63.6)	0.041
Deep vein thrombosis, *n* (%),yes	13 (20.0)	9 (30)	2 (8.3)	2 (18.2)	0.146
Intracranial rebleeding, *n* (%),yes	2 (3.1)	1 (3.3)	1 (4.2)	0 (0)	1
Intracranial infections, *n* (%),yes	0 (0)	0 (0)	0 (0)	0 (0)	1

HRV, Hematoma residual volume; GCS, Glasgow Coma Score; DOH, Duration of hospitalisation; GOS, Glasgow outcome score; ADL, Activities of daily living.

Bold values indicate statistically significant differences.

### Comparison of outcomes and complications during inpatient treatment

**Figure 3:**
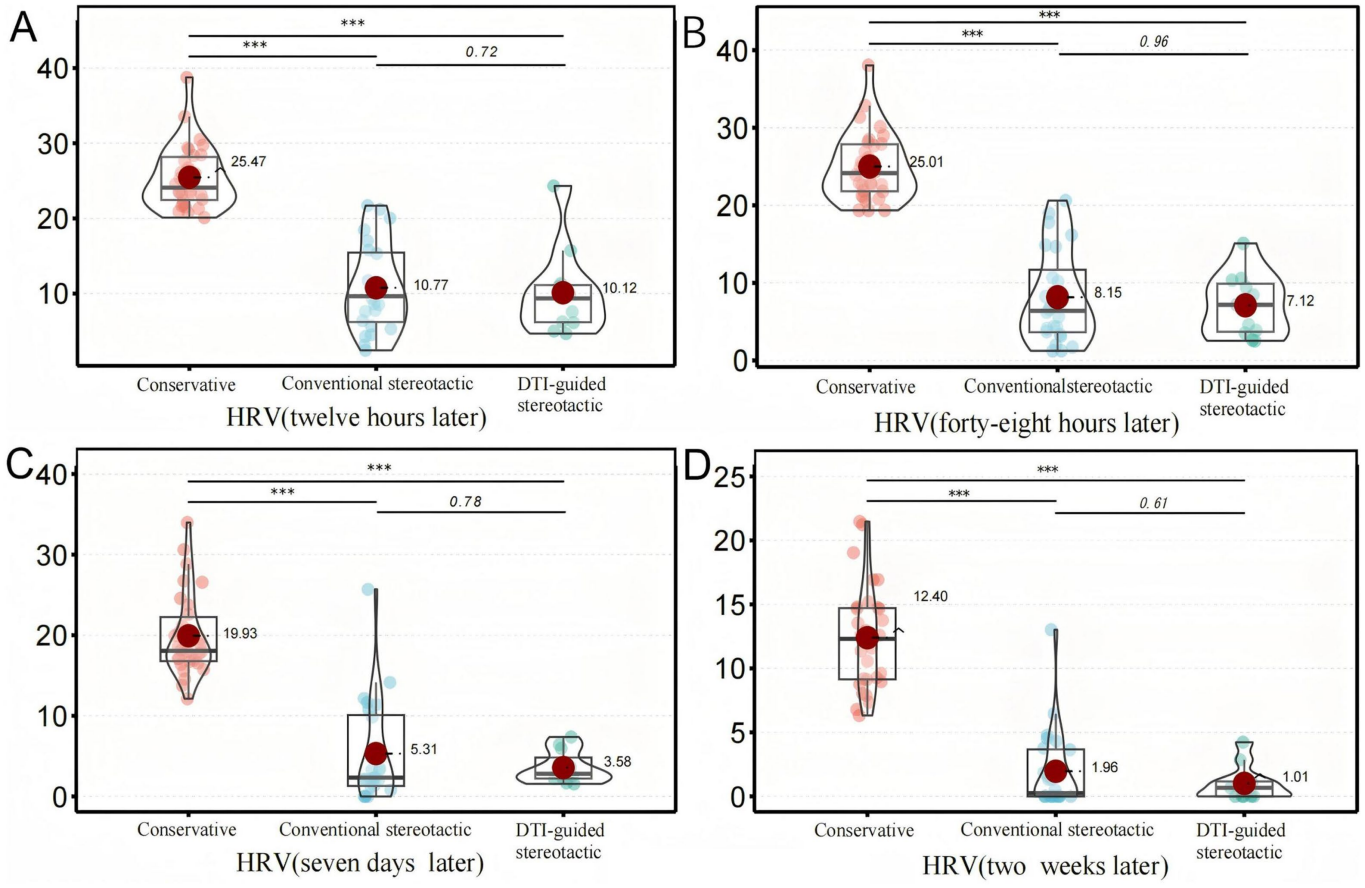
Secondary ending analysis, Figure 3 shows the comparison of HRV among different treatment groups after six months. Panel **(A)** shows the volume changes of HRV in each group after 12 hours of treatment, Panel **(B)** shows the volume changes of HRV in each group after 48 hours of treatment, Panel **(C)** shows the volume changes of HRV in each group after 7 days of treatment, and Panel **(D)** shows the volume changes of HRV in each group after 2 weeks of treatment. The results of the above four figures are presented At 12 hours, 48 hours, 7 days and 2 weeks, the HRV in the conservative group was significantly higher than that in the traditional stereotactic group and the DTI-guided stereotactic group (***p < 0.001).

The study included monitoring of residual hematoma volume at 12 h, 48 h, 7 days and 2 weeks post-treatment for all three groups of patients with repeated head CT scans. The hematoma volume was calculated using the Tada formula (15) and the GCS score was recorded at 2 weeks post-treatment. The results showed that the residual hematoma volume was significantly reduced in both groups A and B compared to the conservative treatment group, with residual volumes of (10.8 ± 5.8 mL) and (10.1 ± 5.7 mL), respectively, after 12 h of treatment compared to (25.5 ± 4.2) ml in the conservative treatment group (*p* < 0.001). The amount of residual hematoma gradually decreased over time and was significantly higher in the conservative group than in groups A and B at all time points (12 h, 48 h, 7d, 2w) (*p* < 0.001). After 2 weeks of treatment, the GCS scores of patients in groups A and B (10.8 ± 2.1) and (11.3 ± 2.3) versus those in the conservative group (11.7 ± 1.8) (*p* = 0.216) were not significantly different between groups, suggesting that the degree of neurological recovery was similar in the short term (Figure 3).

The mean length of hospital stay in each group was compared and the difference was not statistically significant (*p* = 0.425). No intracranial infection occurred during the hospitalisation of the three groups of patients. There was no significant difference between groups in the incidence of pulmonary infection and intracranial rebleeding during treatment in each group (*p* > 0.05), and the incidence of gastrointestinal bleeding in patients in groups a and b was higher than that in the conservative treatment group, with statistically significant differences (*p* < 0.05). The incidence of deep vein thrombosis complications in groups A and B (8.3, 18.2%) was slightly lower than in the conservative treatment group (30%), and the difference was not statistically significant (*p* = 0.146) (Table 2).

### Linear regression analysis affecting ADL scores

Linear univariate regression analysis of ADL score as the primary outcome after 6 months of treatment in all patients showed that there was no statistically significant difference in the effect of sex, age, time from onset to admission, site of bleeding, admission bleeding volume, and history of smoking and alcohol consumption on the prognostic risk of patients (*p* > 0.05). In this study, a potential positive association was found between admission GCS score, hematoma ruptured into ventricle, history of chronic lung disease, serum sodium level and ADL score, with regression coefficients (95% confidence intervals) of 3.49 (0.28, 6.69), 23.33 (9.18, 37.49), 15.67 (0.86, 30.49) and 1.65 (0.09, 3.21), respectively, *p* < 0.05; while there was a potential negative correlation between admission NIHSS score, prothrombin time and ADL score, with regression coefficients (95% CI) of −1.56 (−2.65, −0.47) and −6.47 (−12.66, −0.27), respectively, *p* < 0.05. Regarding treatment modality, group a (*p* = 0.029) and group b (*p* = 0.01) had better long-term recovery of neurological function than the conservative group, and the efficacy of group b was more significant (Table 3).

**Table 3:** Linear univariate regression analysis affecting ADL scores.

Variables	Coeff. (95%CI)	*p* (t-test)	*p* (F-test)
Treatment			
Ref. = Conservative group			**0.015**
Conventional stereotactic	13.37 (1.42,25.33)	**0.029**	
DTI-guided stereotactic	20.36 (4.97,35.76)	**0.01**	
Gender	0.78 (−13.2,14.76)	0.911	0.911
Age	−0.43 (−0.96,0.09)	0.102	0.102
ODTT	−0.05 (−0.99,0.9)	0.922	0.922
Admission GCS	3.49 (0.28,6.69)	**0.033**	**0.033**
Admission NIHSS	−1.56 (−2.65,-0.47)	**0.006**	**0.006**
Hematoma site	4.29 (−7.16,15.74)	0.457	0.457
HRIV	23.33 (9.18,37.49)	**0.002**	**0.002**
BVA	−0.74 (−1.81,0.33)	0.17	0.17
Hypertensive disease	−1.94 (−35.22,31.33)	0.907	0.907
Coronary heart disease	−1.78 (−16.58,13.03)	0.811	0.811
Diabetes	1.44 (−16.05,18.93)	0.87	0.87
COPD	15.67 (0.86,30.49)	**0.038**	**0.038**
Smoking	5.05 (−6.41,16.51)	0.382	0.382
Alcohol consumption	0.3 (−11.51,12.11)	0.96	0.96
Neutrophil	−0.37 (−2.3,1.57)	0.707	0.707
PT	−6.47 (−12.66,-0.27)	**0.041**	**0.041**
APTT	−0.4 (−1.81,1.01)	0.573	0.573
Urea nitrogen	−1.31 (−3.59,0.97)	0.254	0.254
Serum albumin (g/L)	0.2 (−1.22,1.63)	0.779	0.779
Potassium (mmol/L)	−6.25 (−17.64,5.14)	0.277	0.277
Sodium (mmol/L)	1.65 (0.09,3.21)	**0.038**	**0.038**
Calcium (mmol/L)	−7.89 (−28.27,12.49)	0.442	0.442

ODTT, Onset of disease-treatment time; GCS, Glasgow Coma Score; NIHSS, National Institutes of Health Stroke Scale; HRIV, Hematoma ruptured into ventricle; BVA, Bleeding volume on admission; COPD, chronic obstructive pulmonary disease; PT, Prothrombin time; APTT, Activated partial thromboplastin time.

Bold values indicate statistically significant differences.

Subsequent multivariate linear regression analyses were performed with multiple covariate adjustments. The original model indicated that, after 6 months, both the conventional stereotactic group (*β* = 13.37, 95% CI: 1.65–25.1, *p* = 0.029) and the DTI-guided stereotactic group (*β* = 20.36, 95% CI: 5.27–35.46, *p* = 0.01) had higher ADL scores than the conservative treatment group. These differences were found to be statistically significant. Model I was adjusted for gender and age based on the original model. The regression coefficients were *β* = 13.22 (95% CI: 1.52–24.93, *p* = 0.031) for group A and β = 19.72 (95% CI: 4.66–34.77, *p* = 0.013) for group B. The ADL scores remained higher than those of the conservative treatment group, with statistically significant differences observed. Model II was corrected for I + (time from onset to admission, admission GCS and NIHSS scores, site of haemorrhage, admission bleeding volume and rupture of the haematoma into the ventricle). The regression coefficients for groups A and B were *β* = 20.06 (95% CI: 10.68–29.45, *p* < 0.001) and β = 36.51 (95% CI: 23.45–49.58, *p* < 0.001), respectively. After 6 months of treatment, the ADL scores were significantly higher than those of the conservative group. After adjusting Model III for Model II (history of smoking and alcohol consumption, hypertension, coronary artery disease, diabetes mellitus, chronic lung disease, serum leukocyte and neutrophil levels, and serum potassium, sodium, and calcium ion levels), the surgical group (group A) had a β value of 17.82 (95% CI: 6.77–28.86, *p* = 0.003), while group B had a β value of 35.33 (95% CI: 20.2–50.47, *p* < 0.001). After correcting for multiple covariates, the regression coefficients for groups A and B remained significant, with *p*-values of less than 0.05. All the results of the trend tests were *p* < 0.05 and the overall trend results remained stable (see Table 4).

**Table 4:** Linear multifactorial regression analysis of neurological dysfunction in three groups of patients.

Variables	*N*	Original model	*p*	Model I	*p*	Model II	*p*	Model III	*p*
		Crude.β (95CI%)		Adj.β (95CI%)		Adj.β (95CI%)		Adj.β (95CI%)	
Conservative treatment	30	0 (Ref)		0 (Ref)		0 (Ref)		0 (Ref)	
Conventional stereotactic	24	13.37 (1.65~25.1)	0.029	13.22 (1.52~24.93)	0.031	20.06 (10.68~29.45)	<0.001	17.82 (6.77~28.86)	0.003
DTI-guided stereotactic	11	20.36 (5.27~35.46)	0.01	19.72 (4.66~34.77)	0.013	36.51 (23.45~49.58)	<0.001	35.33 (20.2~50.47)	<0.001
Trend testing	65	10.81 (3.66~17.97)	0.004	10.53 (3.4~17.66)	0.005	18.65 (12.48~24.83)	<0.001	17.69 (10.39~24.99)	<0.001

Coefficient is the regression coefficient, 95% CI is the 95% confidence interval, and the corresponding *p*-value (including t-test and F-test). Model I was adjusted for gender and age based on the original model. Model II corrected for I + (time from onset to admission + admission GCS, NIHSS scores + site of haemorrhage + admission bleeding volume + hematoma ruptured into ventricle). Model III adjusted for II + (history of smoking and alcohol consumption + history of hypertension + history of coronary artery disease + history of diabetes mellitus + history of chronic lung disease + serum leukocyte and neutrophil levels + serum potassium, sodium and calcium ion levels).

### Subgroup analysis

**Figure 4:**
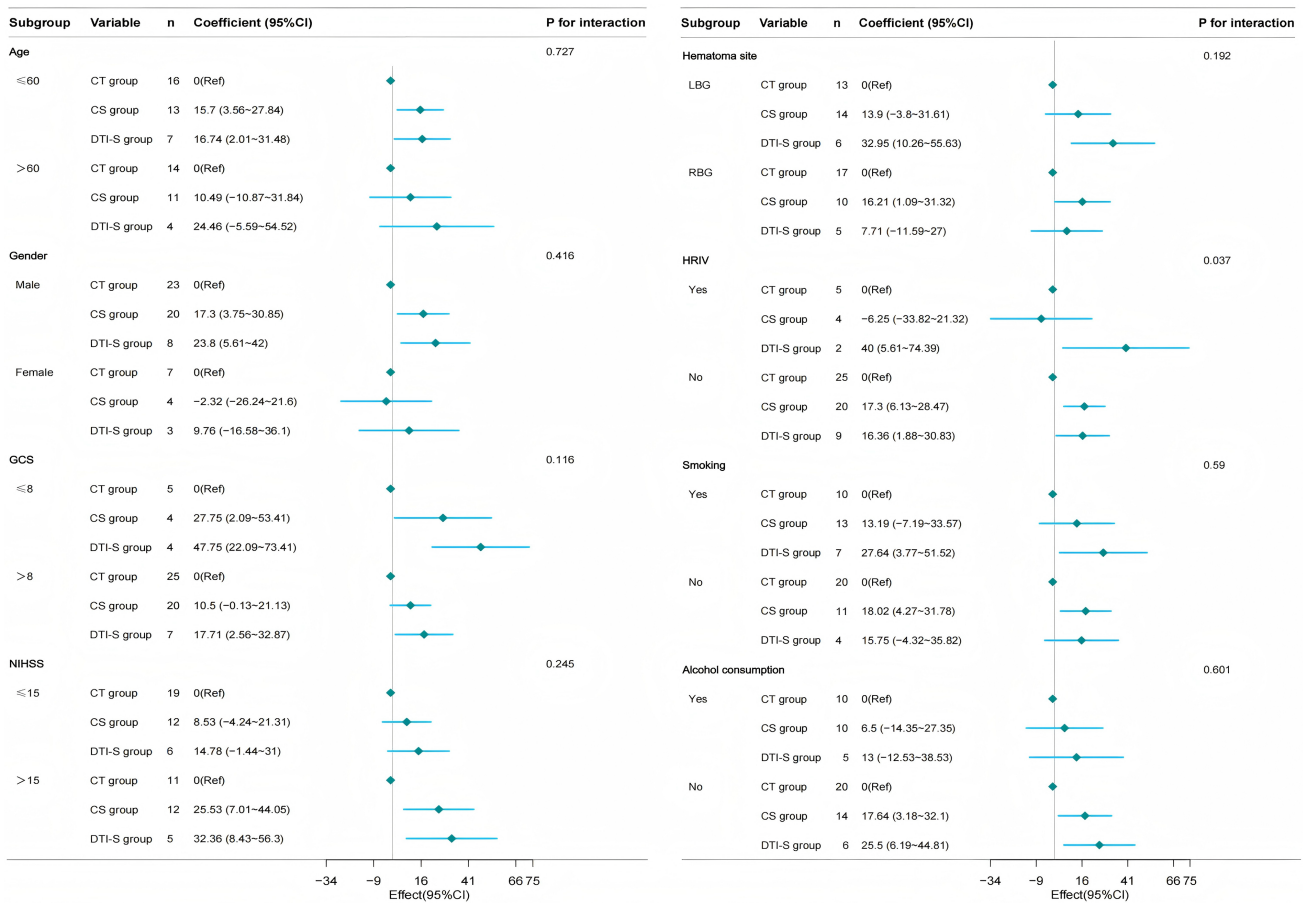
Forest plot showing subgroup analysis. It demonstrates how different subgroup variables (age, gender, GCS, NIHSS, bleeding location, HRIV, smoking and alcohol consumption) impact treatment outcomes. CT group, conservative treatment group; CS group, conventional stereotactic group; DTI-S group, DTI-guided stereotactic; LBG, left basal ganglia; RBG, Right basal ganglia; HRIV, Hematoma ruptured into ventricle.

To further explore the stability of the study results, we also performed subgroup analyses using patients’ ADL scores after 6 months of treatment as the primary outcome. We performed subgroup analyses based on age, gender, GCS score, NIHSS score, site of haemorrhage, hematoma breakthrough into the ventricle, and history of smoking and alcohol consumption. The results showed that conventional stereotactic group and DTI-guided stereotactic group showed a trend towards superiority over the conservative group in most subgroups, but the interactions were not significant in most subgroups (e.g., age, gender, GCS score, NIHSS score, etc., *p* values for the interactions were all >0.05), suggesting that the treatment effect may be relatively consistent across subgroups, supporting the generalisability of the conclusions. Only the interaction effect of hematoma rupture into ventricle subgroup was significant (*P* for interaction<0.05), with a statistically significant difference. The results suggest that essentially the same results were observed in each subgroup as in the overall analysis. There were no significant interactions between the subgroups (Figure 4).

## Discussion

The purpose of this study was to evaluate and compare the efficacy and prognosis of DTI-assisted stereotactic hematoma puncture and drainage surgery, conventional stereotactic hematoma puncture and drainage surgery, and internal conservative management of moderate-volume hypertensive basal ganglia region cerebral haemorrhage. The results suggest that DTI-assisted stereotactic puncture surgery may be a more favourable option for the treatment of patients with moderate-volume hypertensive cerebral haemorrhage of the basal ganglia region because it has a lower perioperative residual hematoma volume, higher scores on activities of daily living, and improved neurological recovery after 6 months of treatment.

Stroke is the second leading cause of death and a major cause of disability worldwide (16, 17). Intracerebral haemorrhage is the second most common subtype of stroke after ischaemic stroke, accounting for approximately 10 to 20% of all strokes (18). Hypertensive cerebral haemorrhage is the most common type of spontaneous cerebral haemorrhage, and the reason for analysing it is that intracranial blood vessels caused by long-term uncontrolled hypertension undergo vitreous and fibrous degeneration, which reduces the elasticity of the blood vessel walls and leads to rupture of the vessel walls and bleeding in the event of a sudden increase in blood pressure (19), and the blood enters the brain tissue and eventually coagulates into a hematoma. Acute basal ganglia haemorrhage tends to damage the corticospinal tract (20), resulting in hemiparesis, cognitive dysfunction and speech impairment. Cerebral haemorrhage can cause primary and secondary injury. Primary injury is mainly due to the rapid accumulation of hematoma in the brain parenchyma, which compresses the surrounding brain tissue, resulting in reduced local cerebral blood flow, midline shift and increased intracranial pressure, which in severe cases can lead to brain herniation (21), while secondary injury may be caused by inflammatory reactions in peripheral nerve tissues or the release of blood components after cerebral haemorrhage, triggering the process of haemoglobin toxicity and inducing cytotoxic oedema and neuronal apoptosis (22, 23). Early hematoma debridement is therefore beneficial because it reduces hematoma volume (24), lowers intracranial pressure, improves cerebral perfusion and reduces secondary neurotoxicity in adjacent brain tissue (25), and timely hematoma removal may improve the prognosis of patients with spontaneous cerebral haemorrhage (26). Stereotactic minimally invasive puncture and drainage (DTI) technique, compared with other surgical methods, can achieve precise localisation and rapid removal of most of the intracranial hematoma through three-dimensional image reconstruction technology, which can effectively alleviate the hematoma-occupying effect and reduce the likelihood of postoperative complications. DTI is an important technique in neurosurgery for guiding and optimising surgical procedures to understand the integrity of nerve fibre bundles in the white matter of the brain (27). The synergistic innovation of DTI and stereotactic techniques has achieved a paradigm shift from ‘anatomical navigation’ to ‘functional protection’.

The main findings of this study indicate that DTI-assisted stereotactic surgical treatment has a better prognosis and quality of life compared to conservative approaches or conventional stereotactic surgery. In addition, this treatment can significantly reduce the amount of residual hematoma in patients after 7d and 2w of treatment and has a high hematoma clearance rate. In terms of pulmonary infection complications, no significant differences were observed between the three groups, and the results were partially consistent with those previously reported in the literature (28). However, the results of this experiment showed that the incidence of gastrointestinal bleeding in patients in both surgical groups was significantly higher than that in the conservative treatment group (*p* < 0.05), which may be due to stress ulcers that occurred as a result of the patient’s body’s stress response after surgery. Although there were no statistically significant differences between the DTI-guided and conventional stereotactic groups for some short-term indicators, the DTI-guided group demonstrated a clear advantage in terms of long-term functional recovery. This suggests that DTI-guided technology could improve long-term patient outcomes, though this needs to be verified by studies with larger sample sizes. In this regard, we also performed a linear multifactorial regression analysis to assess the effect of treatment modality on ADL scores, which showed that both the conventional stereotactic group and the DTI stereotactic group significantly improved ADL scores compared to the conservative group in the different models (both *p* values <0.05), and the effect score of the DTI stereotactic group was higher than that of the conventional stereotactic group in all models, especially in model II (regression coefficient: 36.51 vs. 20.06), representing a stronger and more stable treatment effect in the DTI stereotactic group. In addition, a forest plot was generated to show the interactions between each subgroup, treatment modality, and ADL scores at 6 months. Analysis of the forest plot showed that the individual subgroup trends were generally consistent with the overall trend and positively correlated, indicating that the results were consistently stable. The reason for this may be that in the clinical management of patients with cerebral haemorrhage, the DTI technique can accurately localise the spatial relationship between the hematoma and the CST. A three-dimensional model of the hematoma and the CST is reconstructed to visualise the nerve fibre orientation, and the operator can plan the puncture path and optimise the needle angle to ensure that the puncture trajectory does not intersect the spatial structure of the CST throughout. By avoiding puncture of important functional areas, this solution significantly reduces the risk of mechanical damage to the nerve conduction path during surgery and improves surgical safety. In addition, this surgical method can also rapidly remove hematoma through puncture aspiration and rapidly alleviate nerve damage caused by hematoma (29), which is an important basis for improving the patient’s prognosis and future quality of life. Based on the above, DTI-assisted stereotactic puncture and drainage surgery has outstanding therapeutic advantages. Additionally, significant interactions were observed in the subgroup of haematomas that broke into the ventricles. This may be due to the close anatomical relationship between the ventricular system and surrounding brain tissue. When a haematoma ruptures into a ventricle, it can alter the compression pattern of the surrounding brain tissue and the circulation of cerebrospinal fluid. The surrounding brain tissue undergoes more complex mechanical compression and chemical stimulation in this case. This results in a different pattern of neurological impairment compared to patients whose haematoma does not break into a ventricle. DTI-guided stereotactic surgery can more accurately avoid these complex anatomical structures and areas of damage when planning the penetration pathway. This demonstrates a significant therapeutic advantage in this subgroup. Once a haemorrhage enters the ventricles, blood components can trigger an intense inflammatory response and brain oedema within the ventricular system. This inflammatory response may further exacerbate brain tissue damage and hinder the recovery of neurological function. DTI-guided surgery can remove haematomas more rapidly, reducing the release of inflammatory mediators, thereby playing a greater role in reducing cerebral oedema and improving prognosis.

Although ADL scores showed significant differences between treatment groups, GOS scores did not. This suggests that while DTI-guided stereotactic puncture surgery may improve patients’ ability to perform activities of daily living, it does not improve their overall prognosis. This may be because GOS scores primarily assess patients’ overall functional status, whereas ADL scores more specifically reflect their capabilities in daily living activities. Therefore, future studies need to conduct a more comprehensive assessment of treatment outcomes, including the use of multiple prognostic assessment tools, to more accurately reflect the impact of different treatment methods on patient outcomes. This study has a relatively small sample size, particularly within the DTI group, which comprised only 11 patients. This may limit our ability to accurately estimate differences in treatment efficacy. A small sample size increases the likelihood of random variation in results, which affects the generalisability of conclusions. To address this issue, we plan to conduct a multicentre, prospective study involving a larger patient cohort in future, to more accurately evaluate the long-term efficacy and safety of DTI-guided stereotactic needle biopsy for treating moderate-volume HBGH.

### Limitation

Overall, DTI stereotactic puncture and drainage minimises secondary damage to the CST during surgery. It safely and effectively removes haematomas and stabilises conditions promptly, enabling therapeutic interventions, such as rehabilitation exercises, to be initiated as soon as possible. This could potentially enhance long-term neurological recovery in patients. This difference may be an important factor influencing the results of our study. However, it is important to recognise the limitations of this study. First, because of its retrospective and single-centre design, confounding bias may have been introduced and difficult to mitigate. Second, the sample size of this study was small, particularly in the DTI group. This was primarily due to the fact that this technique is relatively new at our centre, and the patient selection criteria were stringent. This may have affected our statistical power to detect differences in treatment effects, potentially leading to some significant differences going undetected. Additionally, the small sample size may have limited our ability to conduct in-depth analyses of treatment responses in different patient subgroups. Therefore, future studies should be conducted across multiple centres and enrol a larger number of patients in order to evaluate the efficacy of various treatment methods more comprehensively and explore optimal personalised treatment strategies. Finally, selection bias may have occurred in this study because clinicians were unable to maintain a homogeneous standard of care throughout the course of treatment, which may have affected the results. This single-centre retrospective study has a limited sample size and is therefore potentially subject to selection and confounding biases. Although we attempted to control for known confounding factors in our analysis, these biases may still affect the accuracy and generalisability of the results. Therefore, our conclusions require validation through prospective, multicentre studies with larger sample sizes.

## Conclusion

The need for surgical treatment of patients with moderate-volume hypertensive basal ganglia haemorrhage has long been controversial. In this study, we retrospectively analysed and compared the safety and prognosis of DTI stereotactic puncture and drainage, conventional stereotactic puncture and drainage, and internal conservative treatment for cerebral haemorrhage in the basal ganglia region of moderate-volume hypertension. The results of the study showed that patients in the DTI stereotactic surgery group had the highest hematoma clearance rate and the highest scores on the Daily Living Ability Scale at 6 months. Therefore, the long-term prognosis for patients with moderate volume hypertensive basal ganglia haemorrhage may be better. Although the results of this study suggest that DTI-guided stereotactic puncture surgery may offer better long-term outcomes for patients with moderate-volume HBGH, this conclusion requires validation through larger, prospective studies due to the small sample size. Future studies should focus on expanding the sample size to more accurately assess the efficacy of various treatment methods and provide stronger support for clinical decision-making.

## Data Availability

The original contributions presented in the study are included in the article/Supplementary material, further inquiries can be directed to the corresponding authors.

## References

[1] Liu H Hua Y Keep RF Xi G. Brain Ceruloplasmin expression after experimental intracerebral hemorrhage and protection against Iron-induced brain injury. Transl Stroke Res. (2019) 10:112–9. doi: 10.1007/s12975-018-0669-0, PMID: 30315404 PMC6438204

[2] Avan A Digaleh H Di Napoli M Stranges S Behrouz R Shojaeianbabaei G. Socioeconomic status and stroke incidence, prevalence, mortality, and worldwide burden: an ecological analysis from the global burden of disease study 2017. BMC Med. (2019) 17:191. doi: 10.1186/s12916-019-1397-3, PMID: 31647003 PMC6813111

[3] Meretoja A Strbian D Putaala J Curtze S Haapaniemi E Mustanoja S. SMASH-U: a proposal for etiologic classification of intracerebral hemorrhage. Stroke. (2012) 43:2592–7. doi: 10.1161/STROKEAHA.112.661603, PMID: 22858729

[4] Garton ALA Gupta VP Sudesh S Zhou H Christophe BR Connolly ES Jr. The intracerebral hemorrhage score: changing perspectives on mortality and disability. World Neurosurg. (2020) 135:e573–9. doi: 10.1016/j.wneu.2019.12.074, PMID: 31870822

[5] Albakr A AlFajri A Almatar A Aldandan HA Soltan N Ishaque N. Hypertensive intracerebral hemorrhage in young patients from a tertiary care center in Saudi Arabia: an observational study. Prim Care Companion CNS Disord. (2021) 23:20m02768. doi: 10.4088/PCC.20m02768, PMID: 34043888

[6] Wang GQ Li SQ Huang YH Zhang WW Ruan WW Qin JZ. Can minimally invasive puncture and drainage for hypertensive spontaneous basal ganglia intracerebral hemorrhage improve patient outcome: a prospective non-randomized comparative study. Mil Med Res. (2014) 1:10. doi: 10.1186/2054-9369-1-10, PMID: 25722868 PMC4340857

[7] G W M J. Clinical study on minimally invasive liquefaction and drainage of hypertensive putaminal hemorrhage through frontal approach. J Neurol Surg B Skull Base. (2021) 82:258–63. doi: 10.1055/s-0039-1697037 33777641 PMC7987390

[8] Tu WJ Zhao Z Yin P Cao L Zeng J Chen H. Estimated burden of stroke in China in 2020. JAMA Netw Open. (2023) 6:e231455. doi: 10.1001/jamanetworkopen.2023.1455, PMID: 36862407 PMC9982699

[9] Choo YS Chung J Joo JY Kim YB Hong CK. Borderline basal ganglia hemorrhage volume: patient selection for good clinical outcome after stereotactic catheter drainage. J Neurosurg. (2016) 125:1242–8. doi: 10.3171/2015.10.JNS151643, PMID: 26871205

[10] Tang Y Yin F Fu D Gao X Lv Z Li X. Efficacy and safety of minimal invasive surgery treatment in hypertensive intracerebral hemorrhage: a systematic review and meta-analysis. BMC Neurol. (2018) 18:136. doi: 10.1186/s12883-018-1138-9, PMID: 30176811 PMC6120062

[11] Park CH Kim SH Jung HY. Diffusion-tensor-Tractography-based diagnosis for injury of corticospinal tract in a patient with hemiplegia following traumatic brain injury. Diagnostics. (2020) 10:156. doi: 10.3390/diagnostics10030156, PMID: 32183086 PMC7151234

[12] Zhang C Ge H Zhang S Liu D Jiang Z Lan C. Hematoma evacuation via image-guided Para-corticospinal tract approach in patients with spontaneous intracerebral hemorrhage. Neurol Ther. (2021) 10:1001–13. doi: 10.1007/s40120-021-00279-8, PMID: 34515953 PMC8571453

[13] Rehman WA Anwar MS. Surgical outcome of spontaneous supra tentorial intracerebral hemorrhage. Pak J Med Sci. (2017) 33:804–7. doi: 10.12669/pjms.334.12172, PMID: 29067043 PMC5648942

[14] Matsubara M Sonoda S Watanabe M Okuyama Y Okazaki H Okamoto S. ADL outcome of stroke by stroke type and time from onset to admission to a comprehensive inpatient rehabilitation Ward. J Stroke Cerebrovasc Dis. (2021) 30:106110. doi: 10.1016/j.jstrokecerebrovasdis.2021.106110, PMID: 34587577

[15] Xu X Chen X Zhang J Zheng Y Sun G Yu X. Comparison of the Tada formula with software slicer: precise and low-cost method for volume assessment of intracerebral hematoma. Stroke. (2014) 45:3433–5. doi: 10.1161/STROKEAHA.114.007095, PMID: 25316277

[16] Feigin VL. Stroke in developing countries: can the epidemic be stopped and outcomes improved? Lancet Neurol. (2007) 6:94–7. doi: 10.1016/S1474-4422(07)70007-8, PMID: 17239789

[17] O'Donnell M Yusuf S. Tackling the global burden of stroke: the need for large-scale international studies. Lancet Neurol. (2009) 8:306–7. doi: 10.1016/S1474-4422(09)70024-9, PMID: 19233731

[18] Feigin VL Lawes CM Bennett DA Barker-Collo SL Parag V. Worldwide stroke incidence and early case fatality reported in 56 population-based studies: a systematic review. Lancet Neurol. (2009) 8:355–69. doi: 10.1016/S1474-4422(09)70025-0, PMID: 19233729

[19] Pasi M Viswanathan A. Pathophysiology of primary intracerebral hemorrhage: insights into cerebral small vessel disease In: Lee S-H, editor. Stroke Revisited: Hemorrhagic Stroke. Singapore: Springer (2018). 27–46. Available at: https://link.springer.com/chapter/10.1007/978-981-10-1427-7_3#auth-Marco-Pasi

[20] Li J Wei XH Liu YK Chen LS Zhu ZQ Hou SY. Evidence of motor injury due to damaged corticospinal tract following acute hemorrhage in the basal ganglia region. Sci Rep. (2020) 10:16346. doi: 10.1038/s41598-020-73305-8, PMID: 33004960 PMC7530683

[21] Wilkinson DA Pandey AS Thompson BG Keep RF Hua Y Xi G. Injury mechanisms in acute intracerebral hemorrhage. Neuropharmacology. (2018) 134:240–8. doi: 10.1016/j.neuropharm.2017.09.033, PMID: 28947377 PMC6027647

[22] Aronowski J Zhao X. Molecular pathophysiology of cerebral hemorrhage: secondary brain injury. Stroke. (2011) 42:1781–6. doi: 10.1161/STROKEAHA.110.596718, PMID: 21527759 PMC3123894

[23] Lim-Hing K Rincon F. Secondary hematoma expansion and Perihemorrhagic edema after intracerebral hemorrhage: from bench work to practical aspects. Front Neurol. (2017) 8:74. doi: 10.3389/fneur.2017.00074, PMID: 28439253 PMC5383656

[24] LoPresti MA Bruce SS Camacho E Kunchala S Dubois BG Bruce E. Hematoma volume as the major determinant of outcomes after intracerebral hemorrhage. J Neurol Sci. (2014) 345:3–7. doi: 10.1016/j.jns.2014.06.057, PMID: 25034055

[25] Stokum JA Cannarsa GJ Wessell AP Shea P Wenger N Simard JM. When the blood hits your brain: the neurotoxicity of extravasated blood. Int J Mol Sci. (2021) 22:5132 2021 May 12. doi: 10.3390/ijms22105132, PMID: 34066240 PMC8151992

[26] Vespa P Hanley D Betz J Hoffer A Engh J Carter R. ICES (intraoperative stereotactic computed tomography-guided endoscopic surgery) for brain hemorrhage: a multicenter randomized controlled trial. Stroke. (2017) 47:2749–55. doi: 10.1161/STROKEAHA.116.013837 PMC6168060 27758940

[27] Gavin CG Sabin IH. Stereotactic diffusion tensor imaging tractography for gamma knife radiosurgery. J Neurosurg. (2016) 125:139–46. doi: 10.3171/2016.8.GKS161032 27903187

[28] Zhang J Lu S Wang S Zhou N Li G. Comparison and analysis of the efficacy and safety of minimally invasive surgery and craniotomy in the treatment of hypertensive intracerebral hemorrhage. Pak J Med Sci. (2018) 34:578–82. doi: 10.12669/pjms.343.14625, PMID: 30034419 PMC6041508

[29] Huang X Jiang L Chen S Li G Pan W Peng L. Comparison of the curative effect and prognosis of stereotactic drainage and conservative treatment for moderate and small basal ganglia haemorrhage. BMC Neurol. (2021) 21:268. doi: 10.1186/s12883-021-02293-7, PMID: 34229606 PMC8258994

