# Supplementary material for: Treatment and prognosis analysis of patients with moderate-volume hypertensive basal ganglia haemorrhage using DTI-guided stereotactic puncture-based surgery
Source: Front Neurol. 2025 Jul 31;16:1619514. doi: 10.3389/fneur.2025.1619514 (PMC12350388; doi:10.3389/fneur.2025.1619514)

Supplementary figure 1 Designing surgical pathways to visualize ICH and CST with DTI assistance

Supplementary figure 1 Schematic of brain hemorrhage based on DTl fusion. A suggests cerebral hemorrhage and corticospinal tract alignment in the right basal ganglia region, B suggests cerebral hemorrhage and corticospinal tract alignment in the left basal ganglia region; C and D suggest that the patient in Figure A was reviewed after trans-stereotactic paracentesis; E suggests that the patient in Figure B was reviewed after trans-stereotactic paracentesis.

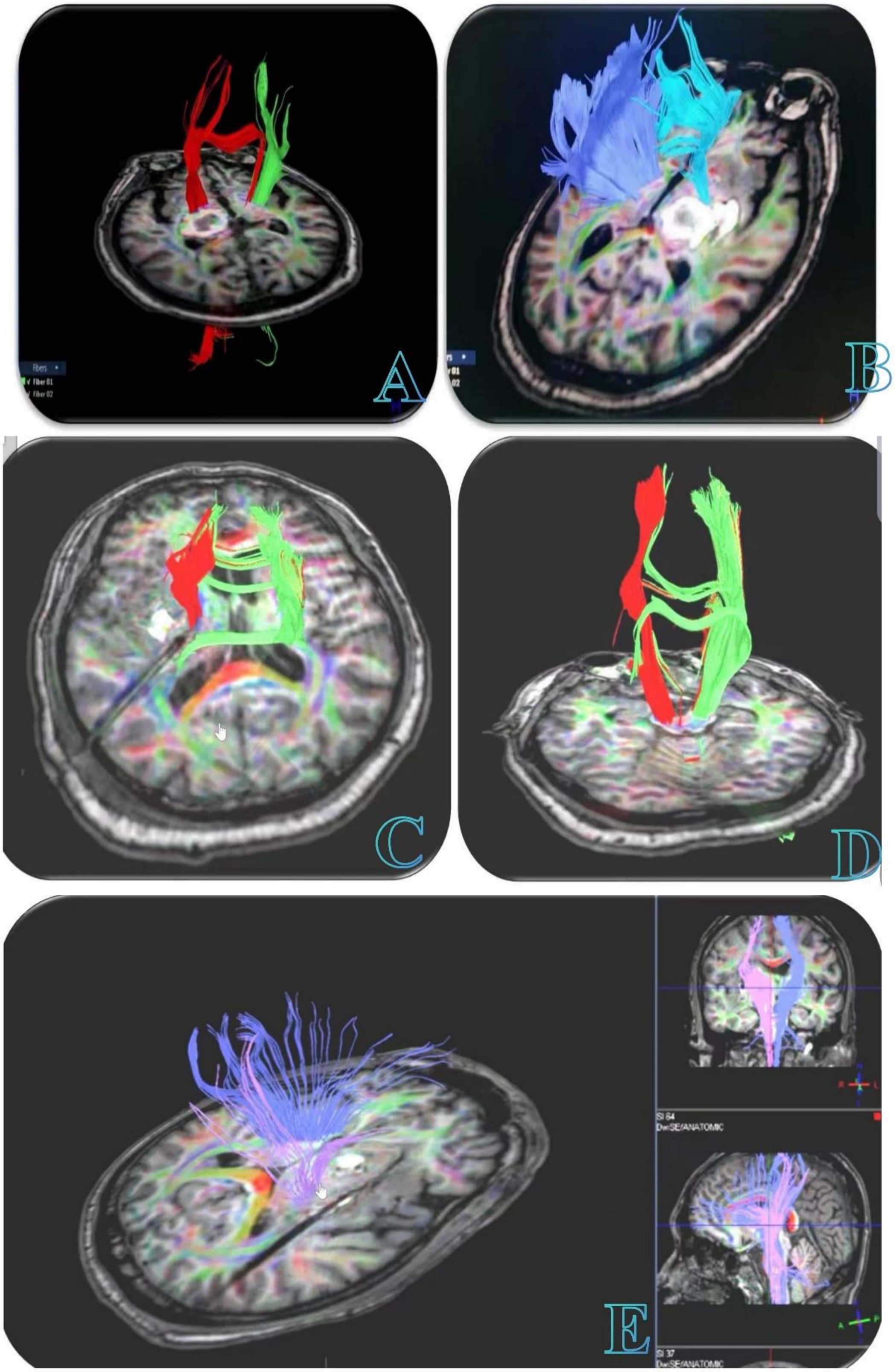

Supplement: Supplementary file 1 [file Image_1.pdf]
